# High glucose promotes macrophage M1 polarization through miR-32/Mef2d/cAMP signaling pathway

**DOI:** 10.1016/j.gendis.2023.03.029

**Published:** 2023-05-03

**Authors:** Cong Chen, Jingsong Cao, Yan Gao, Xiaoping Xie, Zhibo Zhao, Qing Yuan, Yuqi He, Xuyu Zu, Jianghua Liu

**Affiliations:** a The First Affiliated Hospital, Institute of Endocrinology and Metabolism; Center for Clinical Research in Diabetes, Hengyang Medical School, University of South China, Hengyang, Hunan 421001, China; b The First Affiliated Hospital, Department of Laboratory Medicine, Hengyang Medical School, University of South China, Hengyang, Hunan 421001, China; c The First Affiliated Hospital, Institute of Oncology, Hengyang Medical School, University of South China, Hengyang, Hunan 421001, China

Chronic inflammation is a crucial inducer of diabetes vascular complications. One reason is that high glucose easily induces macrophage activation.^1^ Macrophages are the principal participants in innate immunity and exist in all human tissues. In pathological vascular, infiltrated macrophages secrete inflammatory factors leading to an increase in plaque stability.^2^ In macrophage polarization, autophagy plays an important role. Enhancement of macrophage autophagy could induce macrophage polarization from the M1 phenotype to M2 phenotype and inhibit inflammatory reactions.^3^ Our previous research found that high glucose condition promotes miR-32 expression and macrophage M1 polarization,^4^ but the mechanism of miR-32 promoting macrophage M1 polarization is unclear. In this study, we found that, under a high-glucose condition, miR-32/Mef2d/cAMP signaling promoted M1 macrophage polarization via inhibited autophagy. These results provide a theoretical and experimental basis for the prevention and treatment of T2D vascular inflammation.

In this study, because of the difference in glucose concentration between low-glucose DMEM and high-glucose DMEM, mannitol was added to avoid the influence of osmotic pressure on the macrophage. The results showed that the change in osmotic pressure did not markedly affect the expression of autophagy marker genes in macrophages (Fig. S1). Thus, the macrophage was treated with high glucose directly in the following experiments. High glucose condition increased miR-32 expression and promoted macrophage M1 polarization, and inhibited the expression of autophagy marker genes, including sequestosome 1 (p62), autophagy-related 5 (Atg5), autophagy-related 16 like 1 (Atg16l1), and beclin 1 (Becn-1) (Fig. 1A–C; Fig. S2). These results suggested that high glucose promotes macrophage M1 polarization maybe through increased miR-32 expression and inhibited autophagy.

**Figure 1:**
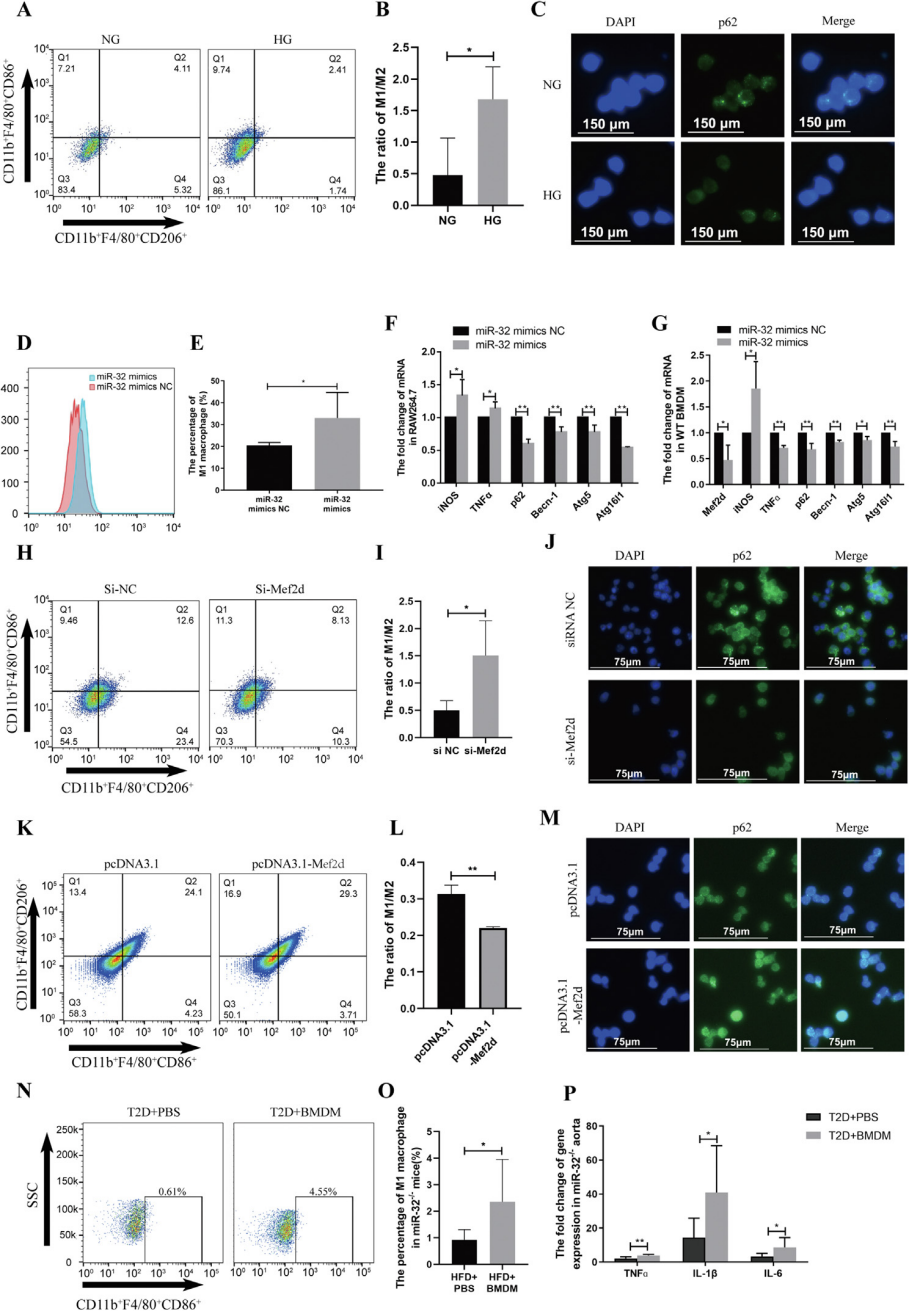
High glucose promoted macrophage M1 polarization via miR-32/Mef2d signaling inhibited autophagy. **(A, B)** Flow cytometry analyzed the influence of high-glucose for macrophage polarization (A); statistical analysis (B). **(C)** Immunofluorescence analyzed the expression of p62 in macrophages after high-glucose treatment. **(D, E)** Flow cytometry analyzed the influence of miR-32 mimics for macrophage M1 polarization (D); statistical analysis (E). **(F)** qRT-PCR analyzed the marker-genes expression of M1 macrophage and autophagy after RAW264.7 transfected miR-32 mimics. **(G)** RT-PCR analyzed the expression of the marker genes of M1 macrophage and autophagy after WT BMDM transfected miR-32 mimics. **(H, I)** Flow cytometry analyzed RAW264.7 M1 polarization after si-Mef2d transfected (H); statistical analysis (I). **(J)** Immunofluorescence analyzed the expression of p62 in macrophage after si-Mef2d was transfected into RAW264.7. **(K, L)** Flow cytometry analyzed RAW264.7 M1 polarization after pcDNA3.1-Mef2d was transfected into RAW264.7. (K); statistical analysis (L). **(M)** Immunofluorescence analyzed the expression of p62 in macrophage after pcDNA3.1-Mef2d was transfected into RAW264.7. **(N, O)** Flow cytometry analyzed the influence of WT BMDM translation for peripheral macrophage M1 polarization in T2D miR-32^−/−^ mice (N); statistical analysis (O). **(P)** qRT-PCR analyzed the influence of WT BMDM translation for aorta inflammation in T2D miR-32^−/−^ mice. ^∗^*P* ≤ 0.05, ^∗∗^*P* ≤ 0.01. Each experiment was repeated more than three times (*n* ≥ 3). The animal experiments were approved by the Animal Welfare and Research Ethics Committee of the Institute of the University of South China.

Then, RAW264.7 and BMDM were used to assess the influence of miR32 on macrophage M1 polarization. After miR-32 mimics were transfected into RAW264.7, macrophage significantly polarized to the M1 subtype by flow cytometry analysis (Fig. 1D, E). qRT-PCR showed that the marker genes of M1 macrophage were all significantly up-regulated, including nitric oxide synthase 2 (iNOS) and tumor necrosis factor alfa (TNFα), while autophagy marker genes were all significantly down-regulated (Fig. 1F). Western blotting also indicated that autophagy was inhibited in RAW264.7 (Fig. S3A, B). Especially, after WT or miR-32^−/−^ BMDMs were transfected with miR-32 mimics, M1 macrophages marker genes were all significantly up-regulated, while autophagy marker genes were all significantly down-regulated (Fig. 1G; Fig. S3C). Importantly, Mef2d was identified as a miR-32 target gene in macrophages (Fig. S3D, E). These results suggested that the inhibition of Mef2d expression and autophagy may be the important pathway for miR-32 promoting macrophage M1 polarization.

Furthermore, to demonstrate Mef2d function in macrophage M1 polarization, RAW264.7 was respectively transfected with si-Mef2d or Mef2d plasmid. After the macrophage was transfected with si-Mef2d, the macrophage significantly polarized to the M1 subtype via flow cytometry analysis (Fig. 1H, I). qRT-PCR found that M1 macrophage marker genes were all significantly up-regulated, while the expression of autophagy marker genes was significantly inhibited (Fig. S4A). Western blotting also indicated that autophagy marker genes were significantly inhibited (Fig. S4B, C). Immunofluorescence found that p62 and Atg5 were all decreased (Fig. 1J; Fig. S4D). Importantly, after RAW264.7 was transfected with the plasmid of pDoubleEx-EGFP-Mef2d or pcDNA3.1-Mef2d, those phenomena were all reversed (Fig. 1K–M; Fig. S5). These findings suggest that Mef2d involves macrophage M1 polarization through regulated autophagy.

In addition, after RAW264.7 cells were co-transfected with the Mef2d plasmid (pDoubleEx-EGFP-Mef2d) and miR-32 mimics, Mef2d and autophagy hallmark genes were all obviously up-regulated at both the mRNA and protein levels (Fig. S6). These findings suggest that Mef2d was the critical gene that mediated the function of miR-32 in macrophage autophagy.

The Mef2d-interacting proteins and autophagy-related genes were searched by Cytoscape software and the GO database, respectively. Between the search results, seven common genes were screened (Fig. S7A). DAVID cluster analysis showed that the Mef2d-interacting protein was involved in 31 signaling pathways. Among them, cAMP, one of the most important secondary messengers involved in autophagy,^5^ was the proper candidate signaling pathway, which includes 3 autophagy genes (Fig. S7B). Furthermore, qRT-PCR verified that the levels of E1A binding protein p300 (Ep300), protein kinase cAMP-dependent catalytic (Prkaca), and mitogen-activated protein kinase 3 (Mapk3) were all increased or decreased after si-Mef2d or Mef2d plasmid transfection (Fig. S7D, E). Therefore, these results indicated that the cAMP pathway was a critical signaling pathway of Mef2d-regulated macrophage autophagy.

*In vivo* experiments were carried out to assess the influence of macrophages on vascular inflammation in miR-32^−/−^ T2D mice. Based on the T2D mouse model,^4^ WT BMDMs were injected into miR-32^−/−^ T2D mice via the caudal vein. Flow cytometer found that WT BMDM injection promoted peripheral macrophage M1 polarization (Fig. 1N, O). qRT-PCR found that the expression of proinflammatory factors (TNFɑ, IL-1β, and IL-6) was significantly increased in the aorta (Fig. 1P). These results suggested that miR-32 BMDM promoted T2D vascular inflammation.

## Author contributions

CC contributed to laboratory experiments, data analysis, and manuscript writing; JC contributed to part of the experimental design and data analysis; YG, XX, and ZZ contributed to part of the experiment; QY and HY contributed to animal feeding; XZ and JL contributed to the guidance of experimental design and manuscript writing. All authors read and approved the final manuscript.

## Conflict of interests

The authors declare that they have no competing interests.

## Funding

This work was supported by the National Natural Science Foundation of China (No. 81873651), Natural Science Foundation of Hunan Province, China (No. 2021JJ40490, 2021JJ70113), and Scientific Research Fund Project of Hunan Provincial Health Commission, China (No. 20201981, 20201901).

## Appendix A Supplementary data

Multimedia component 1 Multimedia component 1

figs1 figs1

figs2 figs2

figs3 figs3

figs4 figs4

figs5 figs5

figs6 figs6

figs7 figs7
