# Supplementary material for: High glucose promotes macrophage M1 polarization through miR-32/Mef2d/cAMP signaling pathway
Source: Genes Dis. 2023 May 3;11(2):539–41. doi: 10.1016/j.gendis.2023.03.029 (PMC10491914; doi:10.1016/j.gendis.2023.03.029)
Supplement: Multimedia component 1 [file mmc1.docx]

**Supplementary Materials and methods**

Ethical approval

This research was approved by the Animal Welfare and Research Ethics Committee of the Institute of University of South China.

STR identification and culture of RAW264.7

RAW264.7 cells were purchased from American Type Culture Collection (ATCC® No. TIB-71™) with a CERTIFICATE OF ANALYSIS in July 2019. RAW264.7 was mycoplasma-free. In May 2022, RAW264.7 was carried out short tandem repeat (STR) Authentication by 18 locus detection. Then, STR results blasted in ExPASy database (Sangon Biotech (Shanghai) Co., Ltd) showed that pairing rate of RAW264.7 cells in ExPASy was 100% (Supplementary material 1).

RAW264.7 cells were cultured in high- or low-glucose Dulbecco’s modified Eagle’s medium (DMEM, Gibco BRL, Grand Island, USA) with 10% fetal calf serum (FBS, Gibco, Australia) and 100 U/ml penicillin-streptomycin at 37 ℃ under 5% CO_2_.

Bone marrow-derived macrophages (BMDM) preparation

Bone marrow cells were obtained from the tibias and femurs of six- to eight-week-old mice. Mononuclear cells were isolated with Mouse Bone Marrow Mononuclear Cell Separation Kit (Solarbio) and cultured in complete DMEM containing 10% heat-inactivated FBS (Gibco), 0.5% penicillin-streptomycin, 10 ng/ml granulocyte-macrophage colony-stimulating factor (GM-CSF) (Beijing Solarbio Science & Technology Co., Ltd., China) and 5 ng/ml macrophage colony stimulating factor (M-CSF) (Solarbio). After culturing at 37 ℃ and 5% CO_2_ overnight, the nonadherent or weakly adherent cells were transferred into another 10 cm plastic dish. Then, the culture medium was changed after 2 days and 5 days. Finally, adherent bone marrow-derived macrophages were collected for further analysis.

Transfection

After the cells were cultured for 24 h and the cell density reached approximately 70%, 1 ml of culture medium was added to the transfection mixture (100 μl of DMEM, 2 μl of Lipofectamine 3000, and 25 nmol of miR-32 mimics, 25 nmol of miR-32 inhibitor, 12 pmol of si-Mef2d or 1 μg of Mef2d recombinant plasmid), which was pre-incubated at room temperature for 20 min. The treated cells were cultured at 37 ℃ under 5% CO_2_ for 24 h or 48 h.

Luciferase assay

The wild-type and mutant sequences of the 3′ untranslated region (UTR) of Mef2d were chemically synthesized and cloned into the pSicheck-2 luciferase reporter plasmid (BGI, China); the constructs were named Mef2d 3′ UTR WT (wild type) and Mef2d 3′ UTR Mut (mutant). Then, 500 ng of Mef2d 3′ UTR WT or Mef2d 3′ UTR Mut and 25 nM miR-32 mimics or NC (RiboBio Co. Ltd.,China) were mixed with Lipofectamine 3000 and then transfected into RAW264.7 cells. The cells were cultured at 37°C under 5% CO_2_ for 48 h. After the cells were harvested, a luciferase assay was performed using the Dual-Luciferase Reporter Assay System Kit (Promega, San Luis Obispo, CA, USA) at Turner BioSystems (Sunnyvale, CA, USA).

Flow cytometry

The samples were divided into three groups: the blank group, the isotype control group and the experimental group. The isotype control group contained with 1 μl of Rat IgG2a Kappa-FITC, 1 μl of Rat IgG2b Kappa-PerCP-Cy5.5, and 1 μl of Rat IgG2a Kappa-APC. In the experimental group, 1 μl of CD86 monoclonal antibody-FITC, 1μl of CD11b monoclonal antibody-PerCP-Cy5.5, and 1 μl of F4/80 monoclonal antibody-APC were added. All groups were incubated at room temperature for 30 min. Then, the cells were resuspended in 1 ml of PBS, concentrated at 250×g for 5 min. Finally, the pellet was resuspended in 300 μl of PBS and detected by BD FACS AriaTM Ⅱ. All flow cytometry antibodies were purchased from Thermo Fisher Scientific Inc.

Immunofluorescence

RAW264.7 was cultured and treated on a highly-adherent slides. The cell fixated with 4% paraformaldehyde for 15 min, and broken membrane with 0.5% Triton for 10min, and blocked with 10% BSA for 30 min. Then, the cells were incubated antibodies overnight and then with secondary antibody conjugated to Alexa Fluor-488 (Absin Bioscience Inc., China). Then, the cells were stained with DAPI (MedChemExpress, NJ). After every step, the cells were washed with PBS 5 min and 3 times. Finally, fluorescence images were observed using the EVOS M7000 (Invitrogen, Carlsbad, CA).

RNA extraction and cDNA synthesis

Total RNA was extracted from cells with an RNA Simple Total RNA Kit (TianGen Biotech (Beijing) Co., Ltd., China). miRNA was extracted with the Mir-XTM miRNA First Strand Synthesis Kit (Beijing Solarbio Science & Technology Co., Ltd., China). cDNA was synthesized by a Revert Aid First Strand cDNA Synthesis Kit (Thermo Fisher Scientific Inc., Waltham, MA USA).

qRT-PCR

20 μl qRT-PCR mixture contained 10 μl of 2 × SYBR Green PCR Mastermix (Takara Biomedical Technology (Beijing) Co., Ltd., China), 1 μl of forward primer, and 1 μl reverse primer (Table 1), 0.2 μl of ROX II (Beijing Solarbio Science & Technology Co., Ltd., China), 1 μl of cDNA template and 6.8 μl of ddH2O. The reaction program was as follows: 95 °C for 2 min, followed by 40 cycles of 95 °C for 15 s and 60 °C for 30 s. The experiment was performed in an ABI 7500.

Cell protein extraction

Cell protein was extracted by Cell Lysis Buffer for Western and IP Kit (Beyotime Biotechnology, Shanghai, China). The protein concentration was detected by a BCA Protein Assay Kit (Solarbio).

Western blotting

After SDS-PAGE, the protein was transferred onto polyvinylidene fluoride (PVDF) membranes by a semidry transfer apparatus (Bio-Rad Laboratories, Inc., USA). The membrane was blocked at room temperature for 1 h with blocking buffer (TBS (Applygen Technologies Inc., China) containing 5% nonfat powdered milk (Sangon Biotech (Shanghai) Co., Ltd., China) and 0.1% Tween-20 (Beijing Solarbio Science & Technology Co., Ltd., China), then incubated with primary antibody overnight at 4 ℃ and washed 3 times for 5 min each time. Next, the membrane was incubated with secondary antibody-linked horseradish peroxidase at room temperature for 40 min and washed 2 times for 20min. Finally, the membrane was developed with an Immobilon Western Chemiluminescent HRP Substrate Kit (EMD Millipore Corporation, Burlington, MA) and analyzed by ChemiDoc ^TM^ XRS+ (Bio-Rad).

Bioinformatics analysis

Cytoscape 3.7.1 software was used to search for the proteins interacting with MEF2D. Then, these proteins were used for gene annotation enrichment analysis (https://david.ncifcrf.gov/summary.jsp). Meanwhile, GO analysis of autophagy-related proteins was performed (http://geneontology.org/), and Venn diagram analysis (https://bioinfogp.cnb.csic.es/tools/venny/index.html) was applied to identify the proteins interacting with Mef2d that was involved in autophagy.

Statistical analysis

All experiments were repeated at least 3 times. The data were analyzed with GraphPad Prism 7.00 software, and the results were shown as mean ± S.E.M. Student’s t test or one-way ANOVA with Bonferroni correction was used to assess statistical significance, and p < 0.05 or p < 0.01 was considered significant or very significant, respectively.

**Supplementary Table**

**Supplementary Table 1 The primers sequence**

| Primer name | Sequence (5′ → 3′) |
| --- | --- |
| miR-32-5p-F* | CGCGCTATTGCACATTACTAAGTTGCA |
| Mef2d-F | GGCTGGCACTAGGCAATGTCAC |
| Mef2d-R | CTGCTGTGGCTGTGGCTGTG |
| p62-F | AGGAGGAGACGATGACTGGACAC |
| p62-R | TTGGTCTGTAGGAGCCTGGTGAG |
| Becn1-F | AGGCAGTGGCGGCTCCTATTC |
| Becn1-R | TGAGGACACCCAGGCAAGACC |
| Atg16l1-F | CAAGCCGAATCTGGACTGTGGATG |
| Atg16l1-R | CGGTCGTGACTTCCTGAGACAATC |
| Atg5-F | TGCGGTTGAGGCTCACTTTATGTC |
| Atg5-R | GTCCCATCCAGAGCTGCTTGTG |
| iNOS-F | ACTCAGCCAAGCCCTCACCTAC |
| iNOS-R | TCCAATCTCTGCCTATCCGTCTCG |
| TNFɑ-F | GCGACGTGGAACTGGCAGAAG |
| TNFɑ-R | GCCACAAGCAGGAATGAGAAGAGG |
| EP300-F | AGCAGTCCACTCCTAGCCTAAGC |
| EP300-R | GGTAGTCCAAGAGCAGCGTAAGC |
| Prkaca-F | GGCTCTCGGAGTCCTCATCTACG |
| Prkaca-R | CGCAGCAGGTCCTTCAAGTCAG |
| Mapk3-F | CTGCTGGACCGGATGTTAACCTTC |
| Mapk3-R | ACTGGCTCATCTGTCGGATCGTAG |
| Gapdh-F | TGTTTCCTCGTCCCGTAG |
| Gapdh-R | CAATCTCCACTTTGCCACT |

* The internal control primers and universal primer of miR-32 were provided by the miRNA Synthesis Kit.

**SupplementaryFigure legend**

**Supplementary Figure 1.** The analysis of osmotic pressure for macrophage autophagy. The experiment repeated more than three times (n≥3).

**Supplementary Figure 2. High-glucose promoted macrophage autophagy**. **(A)** qRT-PCR analyzed the influence of high-glucose for autophagy association-genes. **(B, C)** WB analyzed the influence of high-glucose for autophagy association-genes (B), and statistical analysis (C). **(D)** Immunofluorescence analyzed the expression of Atg5 in macrophage after high-glucose treated. One or two stars respectively represented significant (p≤0.05) or very significant (p≤0.01) change. The experiment repeated more than three times (n≥3).

**Supplementary Figure 3. miR-32 promoted macrophage M1 polarization**. **(A, B)** WB analyzed the expression of Mef2d and autophagy marker-genes after RAW264.7 transfected miR-32 mimics (A), and statistical analysis (B). **(C)** qRT-PCR analyzed the marker-genes expression of M1 macrophage and autophagy after miR-32^-/-^ BMDM transfected miR-32 mimics. **(D)** The WT or mutation sequence of Mef2d 3’-UTR. **(E)** Luciferase assay analyzed the combination of miR-32 and Mef2d 3’-UTR. One or two stars respectively represented significant (p≤0.05) or very significant (p≤0.01) change. The experiment repeated more than three times (n≥3).

**Supplementary Figure 4. The inhibition expression of Mef2d promoted macrophage M1 polarization**. **(A)** qRT-PCR analyzed the marker-genes expression of M1 macrophage and autophagy after si-Mef2d transfected into RAW264.7. **(B, C)** WB analyzed the expression of Mef2d and autophagy marker-genes after si-Mef2d transfected into RAW264.7 (B), and statistical analysis (C). **(F)** Immunofluorescence analyzed the expression of Atg5 in macrophage after si-Mef2d transfected into RAW264.7. One or two stars respectively represented significant (p≤0.05) or very significant (p≤0.01) change. The experiment repeated more than three times (n≥3).

**Supplementary Figure 5. The over-expression of Mef2d inhibited macrophage M1 polarization**. **(A)** qRT-PCR analyzed the marker-genes expression of M1 macrophage and autophagy after pDoubleEx-EGFP-Mef2d transfected into RAW264.7. **(B, C)** WB analyzed the expression of Mef2d and autophagy marker-genes after pDoubleEx-EGFP-Mef2d transfected into RAW264.7 (B), and statistical analysis (C). **(D)** Immunofluorescence analyzed the expression of Atg5 in macrophage after pcDNA3.1- Mef2d transfected into RAW264.7. One or two stars respectively represented significant (p≤0.05) or very significant (p≤0.01) change. The experiment repeated more than three times (n≥3).

**Supplementary Figure 6. Mef2d antagonized the function of miR-32 in macrophage autophagy.** **(A)** qRT-PCR analyzed the antagonism of Mef2d for miR-32 promoted autophagy. **(B, C)** qRT-PCR analyzed the antagonism of Mef2d for miR-32 promoted autophagy (B), and statistical analysis (C). One or two stars respectively represented significant (p≤0.05) or very significant (p≤0.01) change. The experiment repeated more than three times (n≥3).

**Supplementary Figure 7. cAMP signaling is involved in the regulation of Mef2d in autophagy**. **(A)** The search of Mef2d interaction protein involved in autophagy. **(B)** Cluster analysis of the signal pathway regulated by Mef2d interaction protein. **(C)** Mef2d interaction protein involved in cAMP signal pathway, the autophagy associated protein was labeled with red. **(D, E)** qRT-PCR analyzed the expression of autophagy association protein after RAW264.7 transfected with si- Mef2d (D) or pDoubleEx-EGFP-Mef2d (E). One or two stars respectively represented significant (p≤0.05) or very significant (p≤0.01) change. The experiment repeated more than three times (n≥3).
