# Supplementary figures and images for: High glucose promotes macrophage M1 polarization through miR-32/Mef2d/cAMP signaling pathway
Source: Genes Dis. 2023 May 3;11(2):539–41. doi: 10.1016/j.gendis.2023.03.029 (PMC10491914; doi:10.1016/j.gendis.2023.03.029)

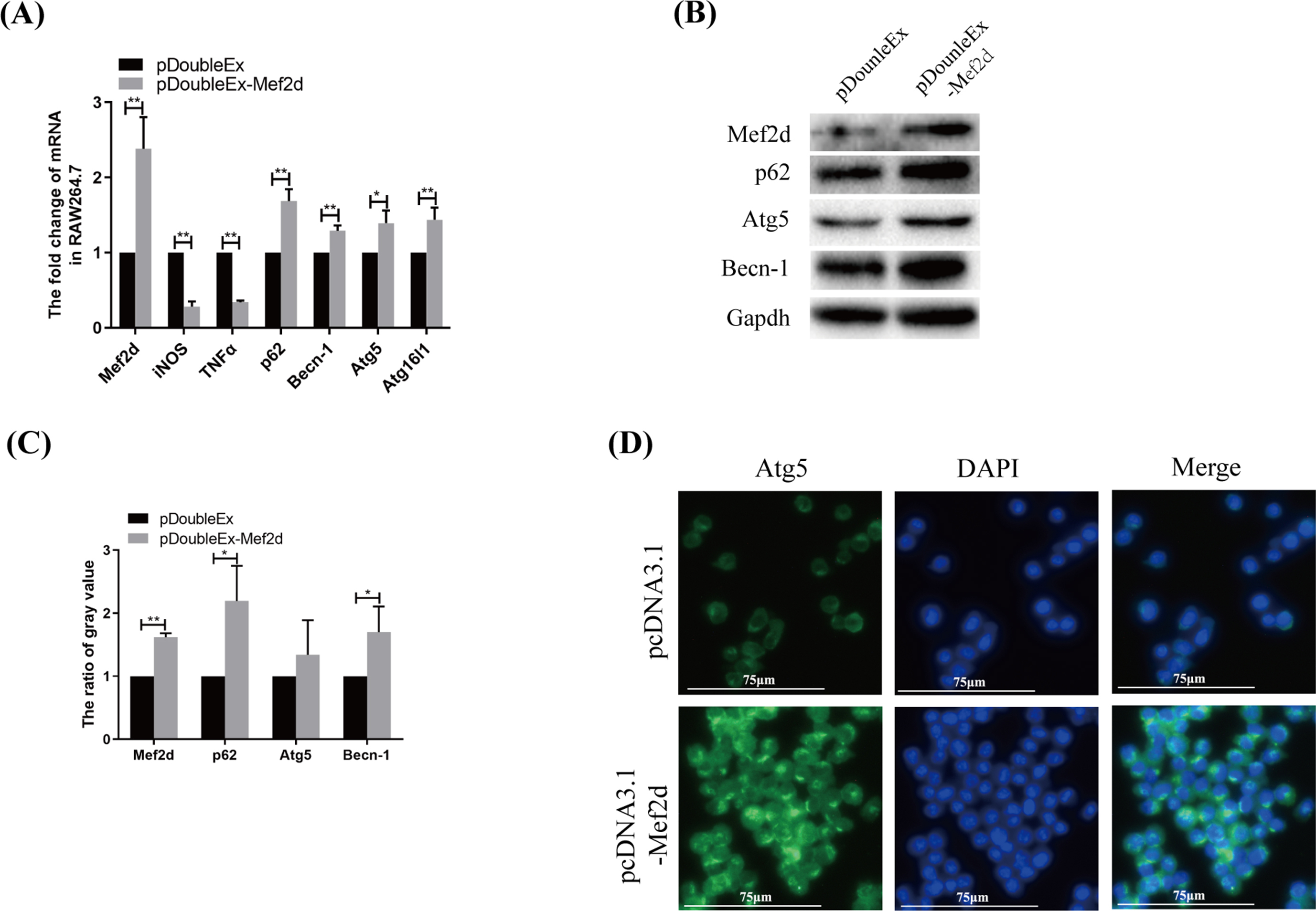

Supplement: figs1 [file figs1.jpg]

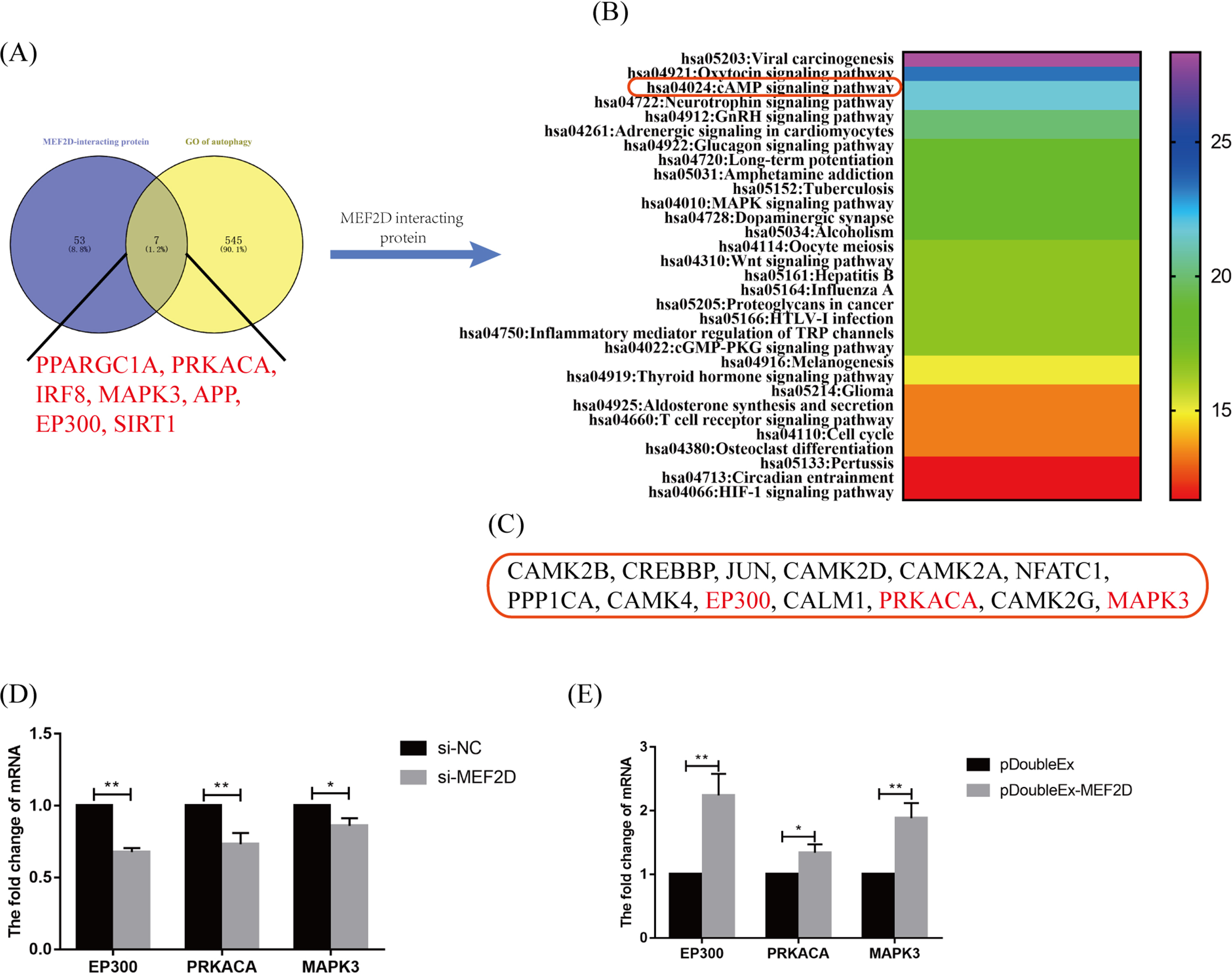

Supplement: figs2 [file figs2.jpg]

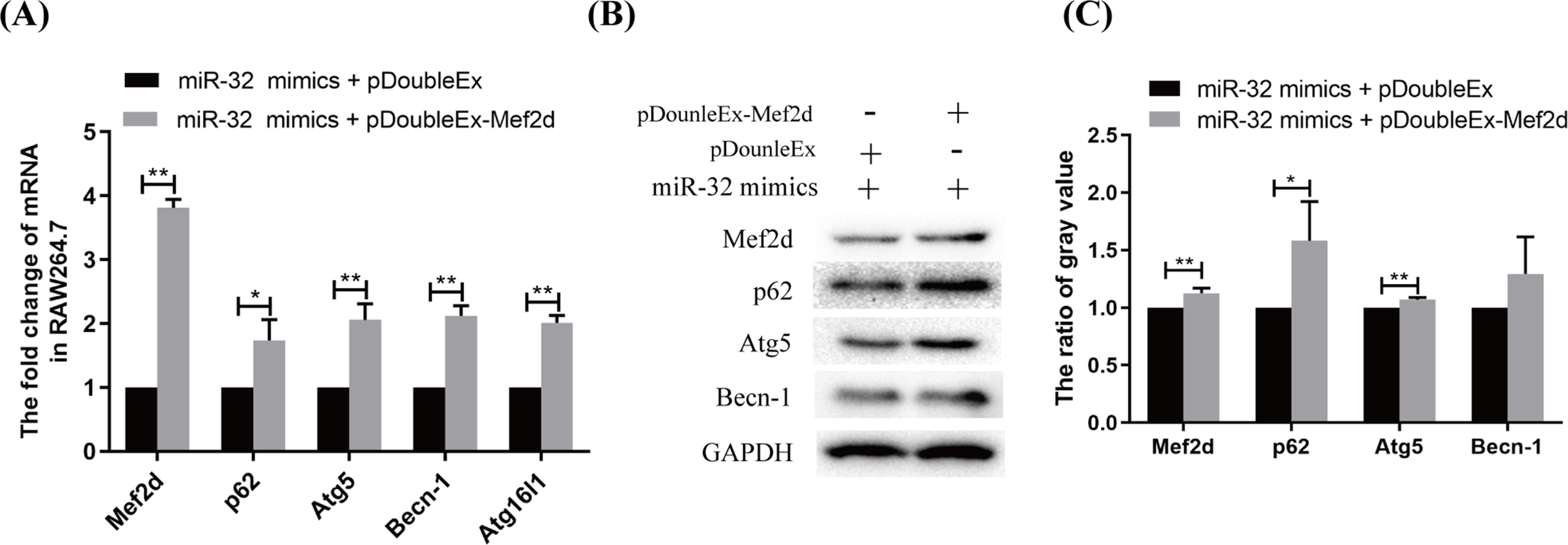

Supplement: figs3 [file figs3.jpg]

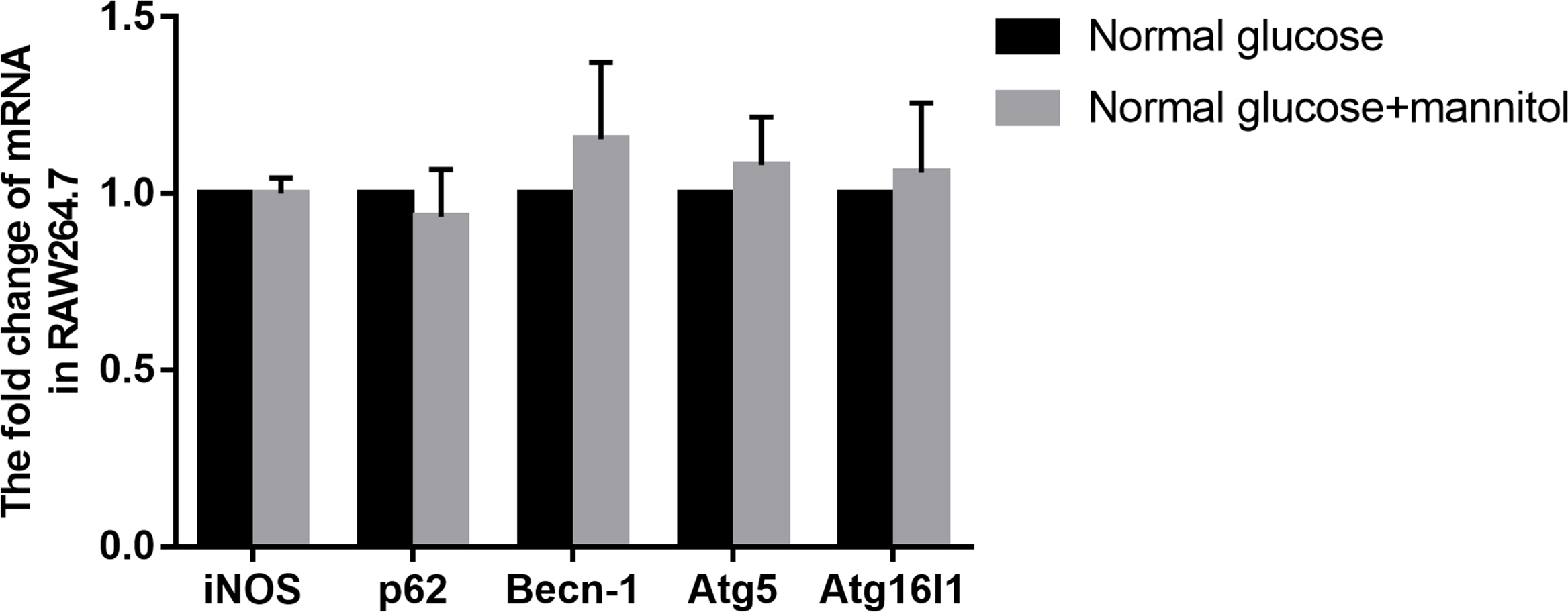

Supplement: figs4 [file figs4.jpg]

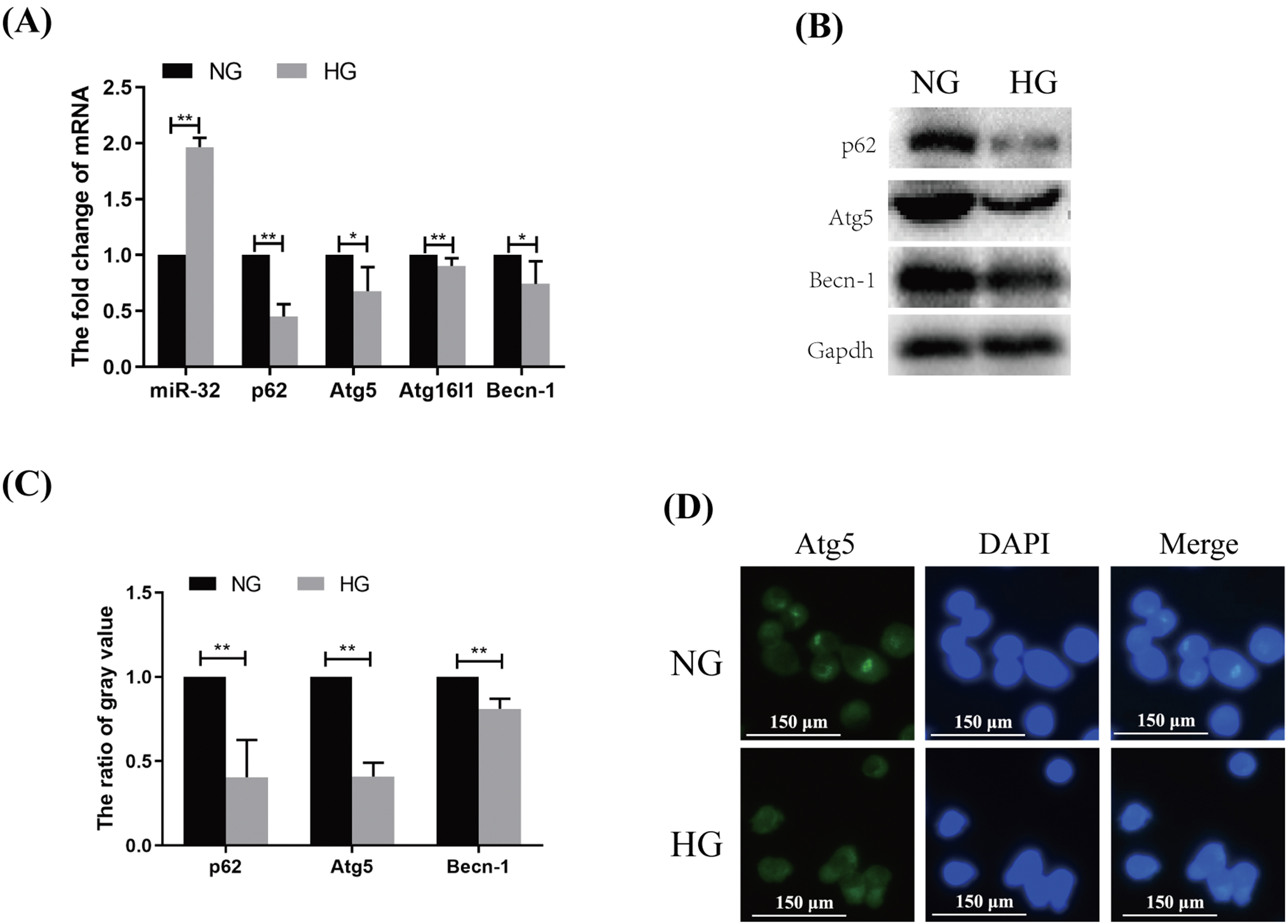

Supplement: figs5 [file figs5.jpg]

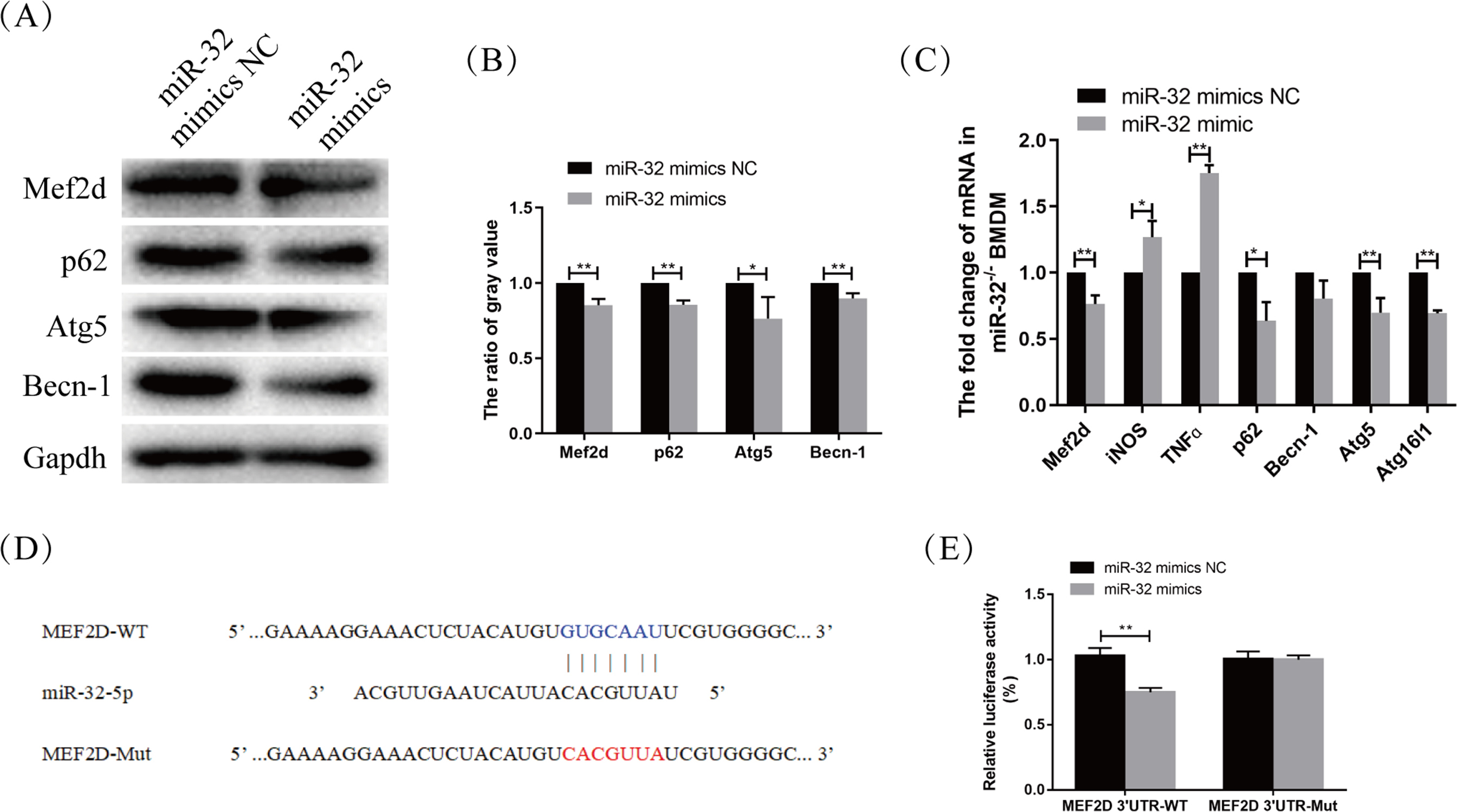

Supplement: figs6 [file figs6.jpg]

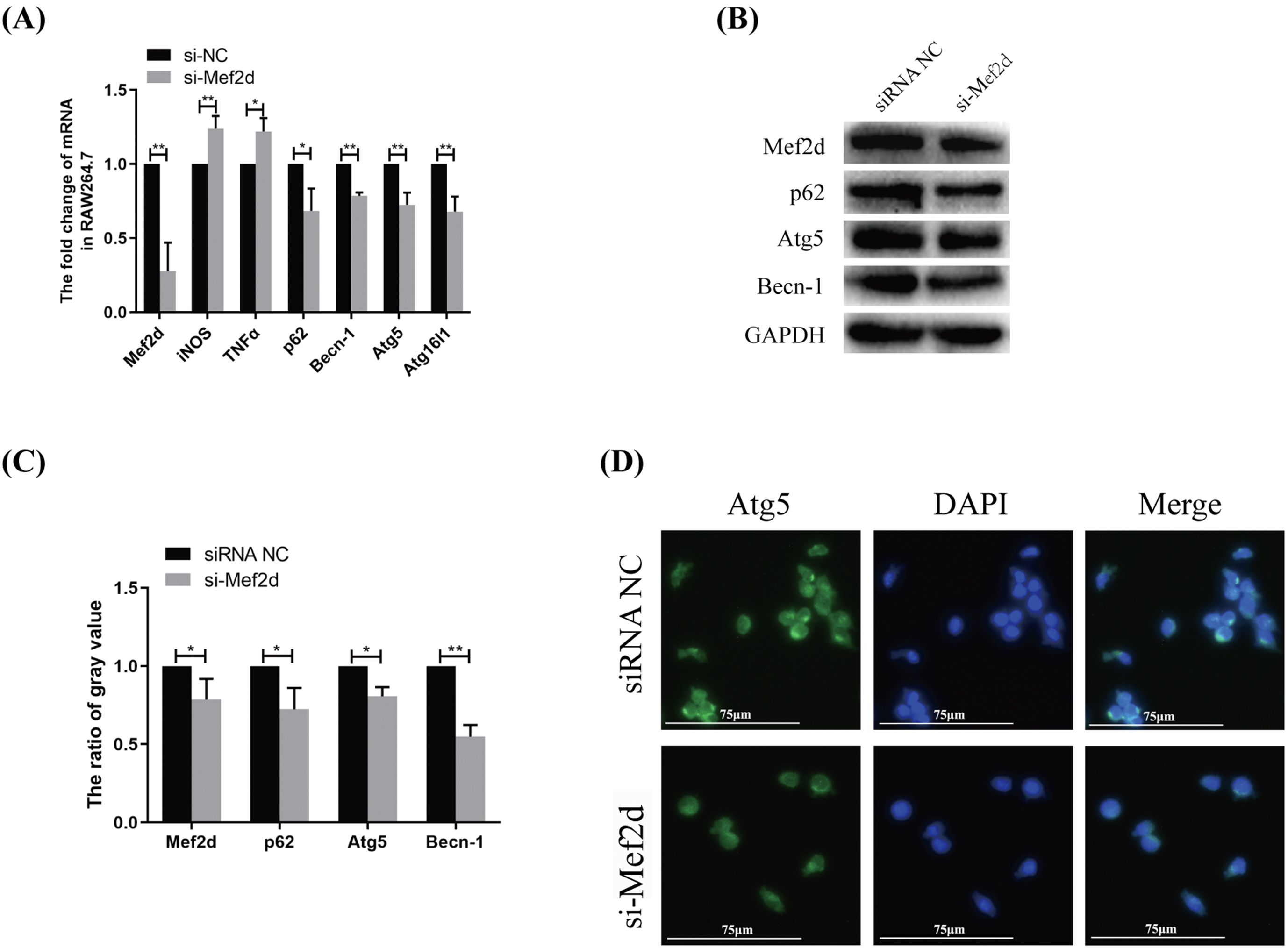

Supplement: figs7 [file figs7.jpg]
